# Editorial: Emerging and reemerging plant viruses in a context of global change

**DOI:** 10.3389/fpls.2022.1108211

**Published:** 2022-12-29

**Authors:** Giuseppe Parrella, Toufic Elbeaino, Paul Leslie Guy

**Affiliations:** ^1^ Institute for Sustainable Plant Protection, National Research Council (IPSP-CNR), Portici, Italy; ^2^ Mediterranean Agronomic Institute of Bari (Centre International de Hautes Études Agronomiques Méditerranéennes (CIHEAM)-Istituto Agronomico Mediterraneo di Bari (IAMB)), Valenzano, Italy; ^3^ Department of Botany, University of Otago, Dunedin, New Zealand

**Keywords:** emerging viruses, climate change, globalization, invasive plants, plant viruses, vector introduction

Emerging and reemerging plant diseases can be defined as those diseases caused by new or reappearing pathogens which, due to their intrinsic characteristics, have the ability to spread rapidly and cause epidemics in certain agro-climatic contexts. In recent years, both DNA and RNA viruses have been implicated in important disease outbreaks in plants. The different forms of mutation, recombination and other types of genetic exchange, considered as the basis of the evolutionary forces of viruses, have undoubtedly given rise tothe genetic diversity found in plant virus populations. In this context, environmental factors play an important role in driving virus evolution. In addition, the rapid expansion of human activity in the world of commerce, agriculture, anthropization of natural ecosystems and climate change have further contributed to the instability between hosts and virus populations, favouring the emergence of viruses with mutant and/or recombinant forms, with potentially negative impacts on plants, vectors and ecosystems.

## Organization of the Research Topic

This Research Topic includes three comprehensive reviews that describe different aspects related to some emerging plant viruses. The first review provides an overview ofnanoviruses (Lal et al.), an important group of emerging viruses with a destructive potential on many crops; capable of causing serious social and economic losses (Johnstone and Mclean, 1987; Hull, 2014). This review deals with nanoviruses features, providing knowledge on their genome organization, replication and their transmission mediated by different aphid species. In addition, the review focuses on the recent emergence of new nanovirus species, often associated with the expansion of their natural host range.

The second review addresses the problem of yellow mottle rice virus (YMRV) (Odongo et al.), currently causing major losses in rice production mainly in sub-Saharan Africa (SSA). This review deals with the molecular characteristics of RYMV: the genomic structure, function and gene diversity, and RYMV-host interactions. In particular, the review sheds light on mechanisms related to the qualitative resistances, controlled by three recessive genes RYMV1, RYMV2 and RYMV3, and quantitative resistances, with the description of several QTLs found in *Oryza* germplasm. Finally, since RYMV shows high genetic variability and some Oryza germplasm has low or non-durable resistance, the review suggests possible genetic improvement strategies for a significant enhancement of rice protection through the use of assisted selection with molecular markers and by employing genome editing to impair susceptibility.

The final review concerns the progress on tomato virome research (Rivarez et al.), with particular reference to the contribution provided by high-throughput sequencing (HTS). The work takes into consideration the period 2011-2020, during which 45 new viruses have been described in tomato, 14 of these identified by HTS. Based on the data present in the literature and in the databases, the authors list 312 viruses, satellite viruses, or viroid species (in 22 families and 39 genera) identified in tomato. This represents the highest number of viral and viral-like agents described in a single botanical species. The work also underlines the importance of the application of HTS for epidemiological studies, in particular for the identification of the virome of weeds and other wild plants, or for the identification of viruses in vectors, irrigation water or wastewater and soil. The HTS analysis of environmental samples helps to greatly improve the understanding of epidemiology and ecology of tomato-infecting viruses and can facilitate virus disease forecasting in order to prevent virus disease outbreaks in tomato. Finally, the review outlines the main tomato viruses, highlighting their potential threat and impact. Newly emerged viruses, such as tomato brown rugose fruit virus (ToBRFV), are capable of overcoming the *Tm*-2^2^ resistance gene used to contain tobacco mosaic virus (TMV) and tomato mosaic virus (ToMV) infections in greenhouse tomatoes. Tomato spotted wilt virus (TSWV) easily overcomes the resistance mediated by the *Tsw* hypersensitivity gene in pepper with increasing temperatures. A group of emerging geminiviruses, of subtropical and tropical origins, including tomato leaf curl New Delhi virus (ToLCNDV) and tomato leaf curl virus (TYLCV), mostly are virulent at high temperatures. Their vector, *Bemisia tabaci*, is notoriously thermophilic and a highly invasive species.

This special issue also contains two articles concerning diagnostic methods for two emerging solanaceous viruses: the first work concerns the setup of loop-mediated isothermal amplification (LAMP) assays, both real time and visual, for the diagnosis of ToBRFV on leaf samples and seeds of tomato and bell pepper (Rizzo et al.). The work also highlights progress in the detection of ToBRFV, mainly regarding sensitivity, compared to previous methods. This paper also emphasizes the practical advantages deriving from the use of LAMP, since it can be applied by poorly equipped laboratories and unskilled persons at official country points of entry, providing a new diagnostic tool for phytosanitary investigations and management of ToBRFV. The second work describes an RT-qPCR assay for the diagnosis of parietaria mottle virus (PMoV), based on a specific TaqMan^®^ probe (Panno et al.),. PMoV is considered an emerging pathogen in the Mediterranean basin on tomatoes and peppers. This virus has recently expanded its natural host range (Parrella et al., 2021). Symptoms on tomato leaves and fruits can be easily confused with those induced by cucumber mosaic virus (CMV) with necrogenic satellite RNA (CMV-satRNA), TSWV or ToMV, and since there are no commercial diagnostic kits available (serological and molecular) on the market, the incidence of PMoV has probably been underestimated up to now in both tomatoes and peppers. Although other molecular diagnostic methods for the detection of PMoV have been previously described and applied for various applications (Parrella, 2020), the diagnostic method proposed in the present work represents an important improvement in the efficiency and applicability of an easily manageable protocol. The improved sensitivity of the method detects 10 copies of ssRNA of PMoV in infected tomato.

The Research Topic also contains eight additional original research works concerning different aspects on some emerging viruses.

The paper of Fiallo-Olivè and Navas-Castillo, identifies the complete genome sequences of two novel virus-satellite complex in *Corchorus siliquosus* collected in Cuba: the begomoviruses corchorus yellow vein Cuba virus (CoYVCUV) and desmodium leaf distortion virus (DesLDD) and two deltasatellite. This is the first report of (i) a monopartite New World begomovirus found in a host other than tomato and (ii) deltasatellites found in *C. siliquosus*, thus extending the host and helper virus ranges of this recently recognized class of DNA satellites.

Tomato chlorosis virus (genus *Crinivirus*, family *Closteroviridae*) (ToCV) is expanding its geographical and host ranges associated with the emergence of whiteflies of the *Bemisia tabaci* complex (Parrella et al., 2014; Bertin et al., 2018). Fortes et al., demonstrated that it is possible to reduce the incidence of ToCV in tomato by selecting for acylsugar-producing *B. tabaci*-resistant tomatoes with type IV glandular trichomes. Candidate resistance genes in melon to the *B. tabaci*-transmitted worldwide emerging begomovirus-ToLCNDV, were identified by Sáez et al., by transcriptome analysis of the resistant WM-7 genotype and the susceptible cultivar Piñonet Piel de Sapo. Transcriptome analysis was also applied by Han et al., to identify responsible host factors for symptom enhancement in the broad bean wilt virus 2 (BBWV2) (genus *Fabavirus* in the family *Comoviridae*), an emerging virus in economically important crops worldwide. They investigated the BBWV2-pepper (*Capsicum annuum* L.) pathosystem, using two distinct BBWV2 strains, PAP1 (a severe strain) and RP1 (a mild strain). Upregulation of several genes associated with pathogen-associated molecular pattern (PAMP)-triggered immunity (PTI) and ethylene signaling were associated only with the severe PAP1 strain, with high ethylene emission detected. Authors conclude that the activation of PTI-associated defense responses increase symptom development during BBWV2 infection in a virus strain-specific manner.

HTS analysis has been applied to study the diversity of virus(es) associated with common bean (*Phaseolus vulgaris* L.) in the North-Western Himalayan region of India by Rashid et al. Three viruses were identified: bean common mosaic virus (BCMV), bean common mosaic necrosis virus (BCMNV), and clover yellow vein virus (ClYVV), with BCMV more widespread and BCMNV and ClYVV new records from India. In another paper (Li et al.), applied HTS to characterize the virome of *Pseudostellaria heterophylla*, generated data on three novel carlaviruses and one novel amalgavirus.

Peng et al., identified and characterized interspecific recombinant viruses between zucchini tigre mosaic virus (ZTMV) and papaya ringspot virus (PRSV) in cucurbits with a novel recombination pattern detected in the HC-pro. Despite the origin from interspecific recombination, they proposed that these viruses still belong to ZTMV according to their genome characteristics; their results provide insights into the prevalence and evolution of ZTMV and PRSV in cucurbits.

The last paper concerns a complementation phenomenon between TYLCV and ToLCNDV-ES, first observed in the field and then demonstrated in the laboratory with agro-inoculations of infectious clones (Vo et al.). ToLCNDV-ES hardly infects tomato and some isolates would not at all. Nonetheless, ToLCNDV-ES in the presence of TYLCV proves to be able to multiply and infect tomatoes. In addition to the known risk of formation of recombinants between geminiviruses, this work highlights the risks of a possible expansion of the natural hosts for this group of emerging viruses thanks to the phenomena of assistance and complementation between viruses.

## Conclusions

In summary, this Research Topic provides cutting-edge methodologies, research, observations and knowledge on the current scenario of *Emerging and Reemerging Viruses in the Context of Global Change.* We are deeply grateful to all the authors and reviewers who with their exceptional work have made possible the realization of this special issue. We believe that this collection will increase knowledge and awareness about the importance of emerging and reemerging viral diseases in order to improve the monitoring and the possible control that derives from them, with the aim to prevent epidemics in agricultural crops.

## Author contributions

All authors listed have made a substantial, direct, and intellectual contribution to the work and approved it for publication.

## References

[B1] Bertin S. Luigi M. Parrella G. Giorgini M. Davino S. Tomassoli L. (2018). Survey of the distribution of *Bemisia tabaci* (Hemiptera: Aleyrodidae) in Lazio region (central italy): a threat for the northward expansion of tomato leaf curl new Delhi virus (*Begomovirus*: Geminiviridae) infection. Phytoparasitica 46, 171–182. doi: 10.1007/s12600-018-0649-7

[B2] Hull R. (2014). Plant virology. 5th edition (London: Academic Press), 854 p.

[B3] Johnstone G. R. Mclean G. D. (1987). Virus diseases of subterranean clover. Ann. Appl. Biol. 110, 421–440. doi: 10.1111/j.1744-7348.1987.tb03274.x

[B4] Parrella G. (2020). Sources of resistance in wild *Solanum* germplasm (section *Lycopersicon*) to parietaria mottle virus, an emerging virus in the Mediterranean basin. Plant Pathol. 69, 1018–1025. doi: 10.1111/ppa.13194

[B5] Parrella G. Nappo A. G. Manco E. Greco B. Giorgini M. (2014). Invasion of the Q2 mitochondrial variant of Mediterranean *Bemisia tabaci* in southern Italy: possible role of bacterial endosymbionts. Pest Manage. Sci. 70, 1514–1523. doi: 10.1002/ps.3686 24272923

[B6] Parrella G. Troiano E. Stinca A. Pozzi M. I. (2021). Molecular and serological detection of parietaria mottle virus in *Phytolacca americana*, a new host of the virus. Phytopathol. Mediterr. 60 (1), 101–104. doi: 10.36253/phyto-12207

